# The Vital Necessity for Fresh Air in Consumption

**Published:** 1890-02

**Authors:** Edmund J. Lee

**Affiliations:** Philadelphia


### THE VITAL NECESSITY FOR FRESH AIR IN CONSUMPTION.

EDMUND J. LEE, M. D., PHILADELPHIA.

Although the literature of consumption is almost limitless, it is often useful to repeatedly call attention to certain necessary facts bearing upon the hygienic treatment of this dreadful disease. Every physician nowadays acknowledges the great importance of proper sanitary conditions in the treatment of this and all other diseases, yet it may be safely affirmed that the majority of physicians do not give this subject the full attention it deserves. It has been said that the three necessities of health, which are free to every one, are the very ones which the vast majority of people use least. These three are *fresh air, exercise*, and *water;* it is needless to mention here the vast use these three necessary things are to the human race.

A comparative few sick people are able to seek hygienic relief by travel; but none are so poor that they cannot properly ventilate their bedrooms or their sick chambers. Yet it is by no means an uncommon experience to enter a sick chamber, even in the houses of the rich, and find the air positively sickening from bad odors. On remonstrating, we are often told the weather is too damp or it is too cold to have a window open; in reality, fresh air is never too damp or too cold not to be preferable to impure, fetid air of a sick room.

In view of these facts, we call attention to a paper read by Dr. Bowditch, on the benefits of short walks in the fresh air; surely this simple and costless expedient can be tried by the poorest or by the busiest. Don't be afraid to go out in "bad weather" provided one is properly clothed. No out-door weather is as bad as the fetid, disease-laden air of a sick room.

### OPEN-AIR TRAVEL IN CONSUMPTION.

Dr. H. I. Bowditch, of Boston, read an interesting paper at the meeting of American Climatological Association to show the great value of "open-air travel as a curer and preventer of consumption, as in the history of a New England family." The family under consideration is that of which the author was a member. At the age of thirty-five his father was undoubtedly threatened with consumption, having cough, hemoptysis, anorexia, diarrhœa, and general malaise, with fever and great debility. In this condition he set out with a friend as his companion and driver, in an open one-horse chaise for a tour through New England. After the first day's travel of twenty-five miles he was so much exhausted and had so much bleeding from the lungs that the friend was advised to carry him home to die. The travelers, however, were both plucky, and kept on, and soon every day's travel brought improved health. In this journey he traveled seven hundred and forty-eight miles, going "down into Rhode Island, thence by the way of Connecticut up through the hills of western Massachusetts to Albany and Troy, and back through Massachusetts to New Hampshire, Vermont, and Maine and then to the home from which he started."

The benefit which he derived from this journey had proved to him the absolute need he had of regular daily exercise in the open air. Afterward, under daily walks of one and a-half to two miles, taken three times daily during thirty years of life, all pulmonary troubles disappeared. He died in 1838, from cancer of the stomach, one lung presenting evidences of an ancient cicatrix at its apex, both being otherwise normal. He was sixty-five years old—*i. e.*, thirty years after the journey. Dr. Bowditch tells us that his father married his cousin, who, after long invalidism, died of chronic consumption in 1834. Notwithstanding the strong predisposing influence to lung disease which would result from such a union, six of their eight children either reached old age or adult life, and were married and have had children and grandchildren, but not a trace of consumption has appeared in any of these ninety-three persons.

This remarkable immunity from consumption Dr. Bowditch attributes to the fact that his father, having experienced in his own case a vast benefit resulting from constant, regular exercise out-of-doors, apparently determined that his children should be early instructed in the same course. Daily walks were required as soon as the children were old enough, and "if any of us, while attending school, were observed to be drooping, or *made the least pretense* even to being not '*exactly well*,' he took us from school, and very often sent us to the country to have farm life and out-of-door play to our heart's content. In consequence of this early instruction all of his descendants have become thoroughly impressed with the advantages of daily walking, of summer vacations in the country, and of camping out, etc., among the mountains. These habits have been transmitted, I think, to his grandchildren in a stronger form, if possible, than he himself had them."

Dr. Bowditch adds: "I submit these facts and thoughts for candid, mature, and *practical consideration* and *use* in the treatment all are called to make of this terrible scourge of all parts of this Union. For my own part, I fully believe that many patients now die from want of this open-air treatment. For years I have directed every consumptive patient to walk daily from three to six miles; *never* to stay all day at home unless a *violent storm* be raging. When they are in doubt about going out, owing to 'bad weather,' I direct them to '*solve the doubt*,' not by staying in the house, *but by going out*."—*Sanitary Inspector*.

